# Laparoscopic Ablation of Hepatocellular Carcinoma in Cirrhotic Patients Unsuitable for Liver Resection or Percutaneous Treatment: A Cohort Study

**DOI:** 10.1371/journal.pone.0057249

**Published:** 2013-02-21

**Authors:** Umberto Cillo, Alessandro Vitale, Davide Dupuis, Stefano Corso, Daniele Neri, Francesco D'Amico, Enrico Gringeri, Fabio Farinati, Valter Vincenzi, Giacomo Zanus

**Affiliations:** 1 Unità di Chirurgia Epatobiliare e Trapianto Epatico, Azienda Ospedaliera Universitaria di Padova, Padua, Italy; 2 Divisione di Gastroenterologia, Azienda Ospedaliera Universitaria di Padova, Padua, Italy; 3 Unità di Medicina Generale, Ospedale San Martino, Belluno, Italy; University of Colorado School of Medicine, United States of America

## Abstract

The aim of this study was to demonstrate the safety and efficacy of laparoscopic ablation for cirrhotic HCC patients. Between January 2004 and December 2009, laparoscopic ablation was applied prospectively in 169 consecutive HCC patients (median age 62 years, 43% hepatitis C positive) considered ineligible for liver resection and/or percutaneous ablation. There was clinically relevant portal hypertension in 72% of cases. A significant proportion of subjects (50%) had multinodular tumors or nodules larger than 25 mm. The main ablation techniques used were radiofrequency in 103 patients (61%), microwave ablation in 8 (5%), and ethanol injection in 58 (34%). The primary endpoint was 3-year survival. There was no perioperative mortality. The overall morbidity rate was 25%. The median postoperative hospital stay was 3 days (range 1–19 days). Patients survived a median 33 months with a 3-year survival rate of 47%. Cox's multivariate analysis identified patient age, presence of diabetes, albumin ≤37 g/l, and alpha-fetoprotein >400 µg/l as significant preoperative predictors of survival, while the chance to undergo liver transplantation and postoperative ascites were the only independent postoperative predictor of survival. Laparoscopic ablation is a safe and effective therapeutic option for selected HCC patients ineligible for liver resection and/or percutaneous ablation.

## Introduction

According to recent guidelines and to the Barcelona Clinic Liver Cancer (BCLC) staging and treatment algorithm [1], surgical resection and percutaneous ablation are the treatments of choice for patients with very early (stage 0) and early (stage A) hepatocellular carcinoma (HCC), but impaired liver function, tumour's location or extension, and patient conditions can strongly limit their applicability. In patients unsuitable for resection and/or percutaneous ablation, the main therapeutic options remain liver transplantation (LT) and transarterial chemoembolization (TACE). However, LT may be indicated for only a minority of HCC patients because of the scarcity of organs [2]. Since LT is generally reserved for patients with a severely impaired liver function (with or without HCC), and given the rising incidence of early HCC diagnoses [3], first-line LT for very early and early HCC patients risks being a theoretical rather than a practical therapeutic option in many countries [4], so a significant proportion of patients with BCLC 0-A HCC judged unsuitable for resection or ablation is offered TACE as the best therapeutic option [4], [5]. It is well known, however, that TACE is only a palliative measure in terms of both survival [1], [6] and its capacity to ensure a genuinely complete histologically confirmed necrosis of the tumour nodules [7]. Its efficacy is also strongly limited by liver decompensation parameters such as ascites, clinically relevant portal hypertension (CRPH), and high bilirubin levels [8].

In this setting, laparoscopic ablation (LA) of liver tumours has the potential to satisfy two fundamental requirements: a) to offer a viable alternative to hepatic resection and/or percutaneous ablation in patients with a BCLC-A HCC; b) to offer a potentially radical therapeutic alternative to TACE in super-selected patients with a BCLC-B tumour. In fact, LA has several potential pathophysiological advantages, making it theoretically suitable for patients with a moderately impaired liver function [9]–[12]. In addition, the opportunity to perform several ablation procedures during the same session, using different techniques in association, also enables the simultaneous treatment of tumours at difficult, multiple and bilobar sites, or of moderately larger dimensions [13]–[15].

This study aims to demonstrate the feasibility, safety and efficacy of LA as first-line therapy for cirrhotic HCC patients considered unsuitable for liver resection and/or percutaneous ablation.

## Materials and Methods

### Ethics statement

The study was approved by the institutional ethics committee at the University Hospital of Padua. Informed consent authorizing storage and use of all relevant data for research purposes was obtained at the time of enrolment as described below. No further authorization was required from our institutional Ethics Committee since the study is a retrospective analysis of prospectively collected data, and only de-identified data were analysed. The Informed Consent is a written consent signed by the patient.

### Patients and variables

**Figure 1:**
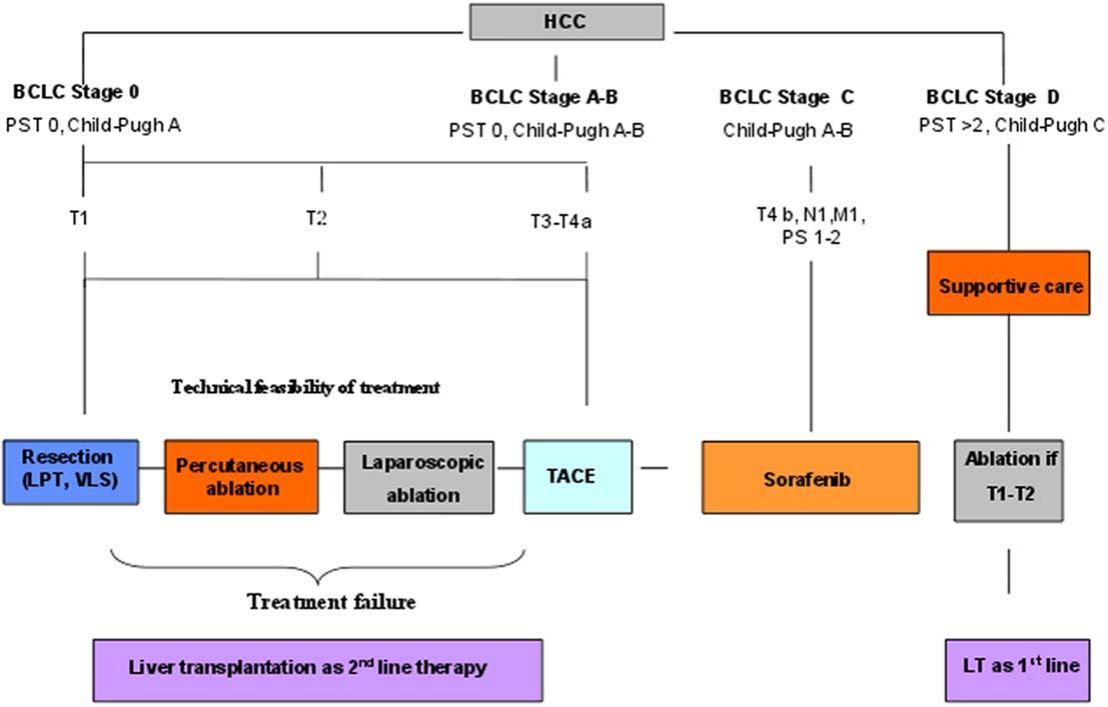
The Padua treatment algorithm for HCC patients.

Consecutive HCC cirrhotic patients evaluated at the Hepatobiliary Surgery and Liver Transplantation Unit at the University Hospital of Padua between January 2004 and December 2009 were treated according to a treatment algorithm (Figure 1) considering LA as first-line therapy for BCLC A patients and super-selected BCLC B patients judged ineligible for liver resection and/or percutaneous ablation due to negative prognostic factors or technical contraindications. Selection criteria for LA are described in detail in Table 1.

**Table 1:** Selection criteria for laparoscopic ablation of HCC patients at the Padua University Hospital.

**INCLUSION CRITERIA**	Ineligibility to liver resection :
	a) Major resection in BCLC A2-A3-A4 patients*
	b) Technical contraindications
	Ineligibility to percutaneous ablation:
	a) critical location (proximity to GI tract or bladder or major hepatic vessels; superficial or exophytic nodules)
	b) tumor extension (size ≥3 cm or ≥3 nodules)
	c) Untreatable ascites
	d) Severe coagulopathy (PT<40% and/or platelets <30×10^9^/l)
**EXCLUSION CRITERIA**	Severe liver decompensation:
	a) MELD >20
	b) Child C class#
	Large multinodular HCC:
	a) Nodule size >7 cm
	b) Number of nodules >5

CRPH, clinically relevant portal hypertension; HCC, hepatocellular carcinoma; GI, gastro intestinal; MELD, model for end stage liver disease.

* AASLD criteria [1] for liver resection were generally followed when tumor location or extension required a major hepatectomy (resection of more than 2 liver segments). In selected cases major liver resection was performed also in HCC patients not following the guidelines [1], using technical expedients such as a porto-caval shunt.

# In selected HCC patients with Child-Pugh C cirrhosis waiting for liver transplantation LA was considered as a bridging therapy: these patients were not included in this study.

Based on our policy (Figure 1), liver transplantation for BCLC A and B HCC patients was only used as second-line or salvage therapy [16], [17].

For all the patients enrolled, the HCC diagnosis was based on AASLD radiological criteria [1], [2] or histology.

Patients were told about the innovative nature of the procedure and their informed consent was a necessary inclusion criterion. All patients underwent a careful preoperative work-up, which included defining the underlying disease according to the Child-Pugh criteria, the MELD score, and morphological study of the tumour using CT and/or MR, and/or contrast-enhanced US, and assessing the anaesthetic risk with the ASA score (*American Society of Anesthesiologists* physical status score).

The data prospectively collected for the study included: the characteristics of the disease and the cirrhotic patients' general status (Child-Pugh score, MELD score, ASA score, liver disease aetiology, presence of oesophageal varices, platelet count); any clinically significant portal hypertension, defined according to the guidelines [1], gastroesophageal varices, splenomegaly with a platelet count below 100,000/ml, or ascites; tumour characteristics (size, location, number of nodules, α-fetoprotein level, and histological grade when available); operative variables (operating time, blood loss, fluid infusion, conversion rate); and postoperative variables (days in hospital, transfusion therapy, ascites leakage from drainage tubes, specific and general complications).

### Surgical procedure and post-operative follow-up

The procedures were performed with patients supine in all cases. The open approach (Hasson's technique) was used to obtain a pneumoperitoneum and the inflation pressure was maintained between 8 and 12 mmHg. A second trocar was inserted in the right or left upper quadrant (for right or left liver lesions, respectively and according to liver anatomy), for the passage of the ultrasound probe. After exploring the peritoneal cavity, laparoscopic intraoperative ultrasound was performed to complete the disease staging, confirm the location, and establish the tumour's relationship with the major hepatic vasculature.

The ablation techniques adopted were radiofrequency (RF) ablation (Cool-tip RF; Valleylab-Tyco Healthcare Group, Boulder, CO, USA), alcohol injection or microwave (MW) ablation (AMICA; HS Hospital Service, Aprilia, LT, Italy).

In the study period RF was the preferred ablation technique. Alcohol injection was mainly used for nodules adjacent to major hepatic vessels or to the gallbladder or intra-abdominal organs. MW ablation was introduced only in the last 12 months of the study period.

Ablation needles were inserted percutaneously and placed inside the lesion under laparoscopic ultrasound guidance. Then a small tubular drain was inserted, to be removed postoperatively.

The local efficacy of ablation at our Institution was evaluated with CT and/or MR performed 20 to 40 days after LA following EASL recommendations as previously reported [17]. In case of complete ablation, we performed an enhanced follow up consisting of CT and/or MR repeated every 3 months for the first year, and every 6 months thereafter. Incomplete ablation or local recurrences were treated according to our treatment algorithm (Figure 1). In this regard, the more common therapies were a repeated LA procedure, TACE, or LT.

### Study design and statistical analysis

This was a retrospective analysis of prospectively collected data.

The primary endpoint was 3-year patient survival. Secondary endpoints were: perioperative mortality (within 30 days after surgery); overall morbidity; postoperative hospital stay (days); leakage of ascites (ml); incidence of early local recurrence (defined as HCC recurrence on the site of ablation within 24 months from LA) . Values for continuous variables were presented as medians (ranges) and values for categorical-nominal variables as frequencies (%). For subgroup comparisons, quantitative variables were compared using Student's *t* or Wilcoxon rank sums tests, and categorical variables were compared using χ^2^ or Fisher's exact tests, as appropriate.

The length of the follow-up after LA was calculated from the date of the operation to the date of the patient's death or latest follow-up. The last follow-up date considered was 15/04/2011. The length of the follow-up and survival were expressed as median (range).

The overall survival curves were calculated using the Kaplan-Meier technique and compared with the log-rank test. Cox's proportional hazards model was used for the univariate analysis to identify the predictors of overall survival after LA. In Cox analyses continuous variables were dichotomized and the median value was considered as the cut-off. The only exception to this rule was α-fetoprotein (AFP), since, as suggested in recent publications [18], [19], it was considered as a dichotomous variable with a cut-off of 400 µg/l independently from their median value in our study cohort. Variables with at least a marginal statistical significance (p<0.1) at univariate analysis were included as covariates in a multivariate Cox model to identify independent survival predictors. We performed two multivariate analyses, one including only preoperative covariates, the other considering both pre and postoperative variables. A p value of less than 0.05 was considered statistically significant.

All statistical calculations were performed using JMP software (1989–2003 SAS Institute Inc.)

## Results

### Patients' characteristics

Laparoscopic ablation was prospectively applied in 169 consecutive HCC patients between January 2004 and December 2009. The characteristics of these patients are shown in Table 2. The sample was a median 62 years of age and males predominated. The main aetiology of cirrhosis was hepatitis C virus infection (43%), followed by alcohol abuse (30%). Clinically relevant portal hypertension was recorded in 72% of cases. One hundred and three patients (61%) had new-onset HCC and there appeared to be a single lesion in 85 cases (50%). Most patients (67%) were in stage BCLC 0-A, but we had only a small proportion of patients in stages BCLC 0, A1, and A2 (17%) in our series (Table 2). Histological grading was available for 104 patients (based on biopsy of the nodule in ablated patients); 6 patients (6%) had a poorly-differentiated tumour.

**Table 2:** Clinicopathological characteristics.

PATIENTS' CHARACTERISTICS	
Age (years)	62 (34–84)
Males	143 (85%)
BMI	26 (18–41)
ASA score >2	76 (45%)
Diabetes	49 (29%)
Etiology	
HCV	72 (43%)
HBV	34 (20%)
Exotoxic	50 (30%)
Other	13 (7%)
Clinically relevant portal hypertension	121 (72%)
Child-Pugh B	57 (34%)
Platelets (10^9^/l)	96 (14–334)
Albumin (g/l)	37 (23–56.8)
Total bilirubin (µmol/l)	22.3 (4.9–175.8)
INR	1.2 (1–1.6)
Creatinine (µmol/l)	78 (45–236)
Na+ (mmol/l)	139 (125–146)
MELD	10 (6–21)
New-onset HCC	103 (61%)
Alpha fetoprotein (µg/l)	23 (1–28356)
Alpha fetoprotein >400 µg/l	17 (10%)
Diameter of the largest nodule (mm)	25 (10–68)
Number	
1	85 (50%)
2–3	61 (36%)
>3	23 (14%)
BCLC-A2	
0	6 (3%)
A1	17 (10%)
A2	7 (4%)
A3	45 (27%)
A4	37 (22%)
B	57 (34%)
Ablation procedure (Total)	169
RF	103 (61%)
Alcohol	58 (34%)
MW	8 (5%)
Transplantation as 2^nd^ line therapy	33 (20%)

ASA, American Society of Anesthesiologists physical status score; HCV, hepatitis C virus; HBV, hepatitis B virus; MELD, model end-stage liver disease score; HCC, hepatocellular carcinoma; BCLC, Barcelona Clinic liver cancer stage; RF, radiofrequency; MW, microwave.

For most patients, the main ablation technique was RF, performed in 103 patients (61%), MW ablation was used in 8 (5%), and ethanol injection alone in 58 (34%).

### Secondary endpoints

The median operating time was 100 minutes (range 40–220 minutes). Plasma transfusions were given to 16 patients (9%), while blood loss higher than 100 ml occurred in only 9 cases (5%). The conversion rate during LA was 2% and this occurred for intra-peritoneal adhesions in 2 cases and for intraoperative bleeding difficult to control laparoscopically and requiring blood transfusion on only one occasion.

There was no perioperative mortality. The overall morbidity rate was 25%. The most common postoperative complication was ascites, occurring in 32 patients (19%), defined for the purposes of this study as a leakage from abdominal drains exceeding 1000 ml/day during the hospital stay. LA-treated patients developed fever in 12% of cases and renal failure in 2%. Encephalopathy, anaemia, spontaneous bacterial peritonitis occurred less often. The median postoperative hospital stay was 3 days (range, 1–19 days).

Only 15% of patients had incomplete LA at first imaging study. Incidence of early local recurrence was 28%. However, other 24% of enrolled patients had distant recurrences during the study period.

### Survival analysis

**Figure 2:**
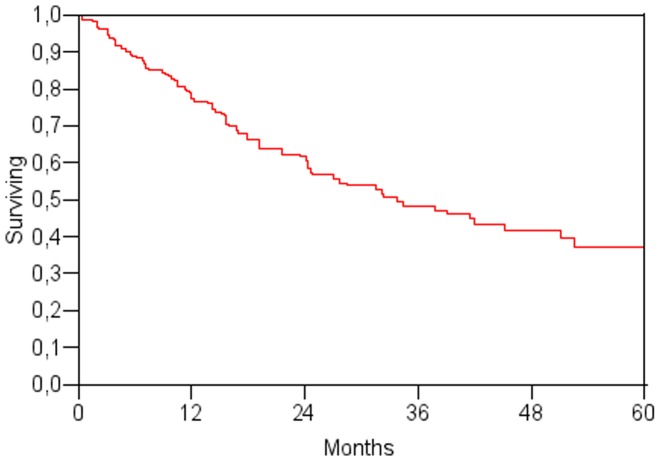
Overall survival curve.

Patients survived a median 33 months, and overall survival at 1, 3, and 5 years was 79%, 49%, and 40% respectively (Figure 2). The median follow-up for survivors was 30 months (range 12 to 78 months). Eighty-two (49%) of patients died during the study period. The main cause of death was tumor progression (73%), but a consistent proportion of patients (27%) died due to liver decompensation.

Due to incomplete LA, tumour recurrence, or liver function impairment about half of enrolled patients (57%) had other therapies during their follow-up: other laparoscopic procedures in 30 cases (18%), percutaneous ablation in 18 patients (11%), ablation and/or TACE in 15 (9%), liver transplantation as second-line therapy (Figure 1) in 33 patients (19%).

All the variables in Table 2 were considered for the survival analysis, together with postoperative covariates such as operating time, hospital stay, morbidity, plasma or blood transfusions, postoperative ascites, other therapies during follow up, and liver transplantation.

**Figure 3:**
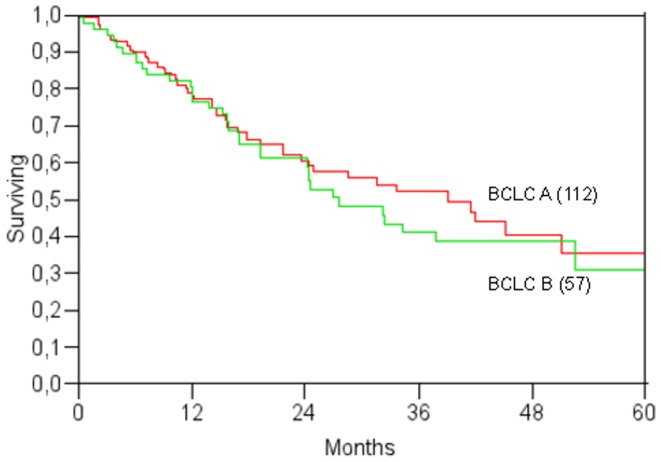
Kaplan-Meier survival curves by BCLC stage (p>0.05).

Table 3 lists the variables having a significant or marginal impact on survival (p<0.1) at Cox's univariate analysis. The only significant tumour-related variable was a serum level of α-fetoprotein >400 µg/l. BCLC staging did not have a relevant prognostic value in our series (Figure 3). The median survival of BCLC-A and BCLC-B patients were 39 and 28 months, respectively, and the difference was not statistically significant.

**Table 3:** Univariate survival analysis.

	Hazard ratio (95% confidence interval)	*p* value
Age >62 years	1.43 (1.14–1.80)	0.0015
ASA >2	1.39 (1.11–1.74)	0.0042
Diabetes	1.31 (1.04–1.64)	0.0248
Albumin ≤37 g/l	2.02 (1.28–3.20)	0.0023
Child-Pugh B	1.75 (1.11–2.73)	0.0160
Alpha fetoprotein >400 µg/l	1.68 (1.25–2.19)	0.0010
Postoperative ascites^1^	1.55 (1.18–1.99)	0.0020
Postoperative complications	1.68 (1.00–2.73)	0.0491
No liver transplantation	1.86 (1.33–2.80)	0.0001

1 leakage from abdominal drains >1000 ml/day.

ASA, American Society of Anesthesiologists physical status score; BCLC, Barcelona Clinic liver cancer stage.

Other variables capable of predicting survival related to patients' general conditions (age, diabetes, ASA score), liver function (hypoalbuminemia, Child Pugh B class), or postoperative course (postoperative ascites, postoperative complications and liver transplantation). Interestingly portal hypertension, MELD score and other liver function tests had a poor prognostic performance in this study. The histological grade variable was not included in the analysis because it was only available for 104 patients.

**Figure 4:**
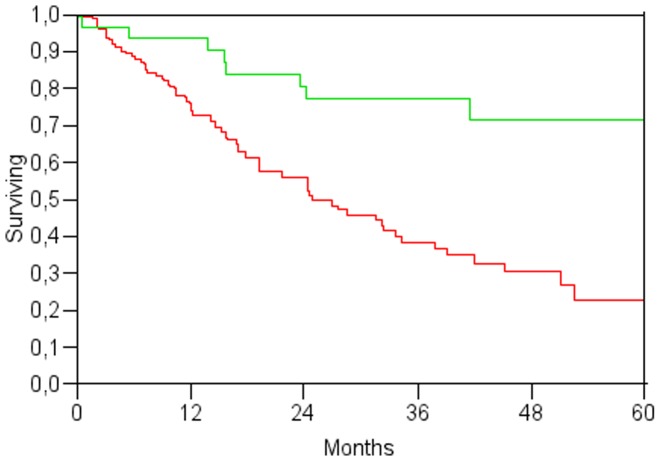
Survival according to possibility to have liver transplantation after LA (p = 0.0004).

Two multivariate analysis were performed (Table 4): the first including only preoperative variables identified age, AFP >400 µg/l and albumin ≤37 g/l as significant predictors; the second including both pre- and the postoperative variables identified diabetes, AFP >400 µg/l and albumin ≤37 g/l as preoperative independent predictors of survival, while postoperative ascites and the opportunity to undergo liver transplantation (Figure 4) were the sole postoperative predictors. ASA score showed only a marginal impact on survival at multivariate analysis.

**Table 4:** Multivariate survival analyses.

	Hazard ratio (95% confidence interval)	*p* value
Model including only pre-operative covariates		
Age >62 years	1.47 (1.14–1.91)	0.0028
ASA >2	1.21 (0.93–1.58)	0.1455
Diabetes	1.28 (0.99–1.65)	0.0614
Albumin ≤37 g/l	2.99 (1.70–5.32)	0.0001
Child Pugh B	0.81 (0.47–1.40)	0.4474
Alpha fetoprotein >400 µg/l	2.17 (1.56–2.96)	<0.0001
Model including pre and post-operative covariates		
Age >62 years	1.28 (0.89–1.57)	0.2581
ASA >2	1.30 (0.99–1.69)	0.0584
Diabetes	1.37 (1.03–1.82)	0.0308
Albumin ≤37 g/l	2.80 (1.58–5.01)	0.0004
Child Pugh B	0.75 (0.43–1.31)	0.3131
Alpha fetoprotein >400 µg/l	2.10 (1.50–2.89)	<0.0001
Postoperative ascites^1^	1.60 (1.12–2.26)	0.0109
Postoperative complications	0.73 (0.36–1.44)	0.3646
No liver transplantation	2.07 (1.36–3.44)	0.0004

1 leakage from abdominal drains >1000 ml/day.

ASA, American Society of Anesthesiologists physical status score; BCLC, Barcelona Clinic liver cancer stage.

## Discussion

To the best of our knowledge, this study represents the largest single-centre series on laparoscopic ablation for HCC on cirrhosis published to date [9]–[15], [20]–[30]. Our results broadly overlap with reports from other study groups, particularly concerning the safety of the procedure, with no perioperative mortality and a low incidence of specific morbidity (25%), which is comparable with other experiences [10] [21] [24] [26]; our conversion rate (2%) was also in line with the literature [14]. Our median postoperative hospital stay was relatively short (3 days) and similar to that of other experiences [27] [–30].

In other experiences [21], however, LA was used in patients eligible for traditional treatments, after selecting patients according to the number and size of their nodules and the severity of their cirrhosis. Our enrolment criteria were particular in that we prospectively applied LA super-selecting patients considered unresectable or ineligible for percutaneous ablation.

The particular feature of our treatment algorithm (Figure 1) lay in considering a sort of “hierarchy” of therapeutic options assigning whenever possible a potentially radical therapy regardless of BCLC stage: the first option to consider was liver resection or percutaneous ablation, the second laparoscopic ablation in those for whom resection or percutaneous ablation were technically infeasible, while TACE and Sorafenib were only considered when the previous where judged infeasible due to tumour stage (Table 1).

Due to the shortage of organs in Italy and the strong epidemiological pressure of HCC [3], we considered liver transplantation as the first-line option only for Child C patients with HCC [31] or young HBV positive patients with multinodular HCC, while it was considered only for second-line therapy in patients after the first-line options failed for recurrence or incomplete treatment.

This enrolment policy enabled LA to be offered as a viable alternative to resection and/or percutaneous ablation for patients with stage BCLC-A, and as a potentially radical therapeutic alternative to TACE for super-selected patients with stage BCLC-B.

The role of LA in our treatment algorithm is justified by some theoretical advantages of the laparoscopic approach with respect to the percutaneous procedures which include the ability to approach lesions adjacent to the gastrointestinal tract, gallbladder and bile ducts, or in presence of thrombocytopenia and the chance to perform intraoperative ultrasound for a more accurate targeting of the lesions. In addition, the related pneumoperitoneum allows for an up to 40% reduction in the portal venous flow, thereby enabling an increase in the size of the ablation site; and the general anaesthesia means that a higher dose of radiofrequency energy can be used, or a larger amount of absolute alcohol can be injected in the case of alcoholics, by comparison with the percutaneous approach [9] [–12] [18].

In the present study, enrolled patients survived a median 34 months, while the median survival of BCLC-A and BCLC-B patients were 39 and 28 months, respectively.

Untreated patients with intermediate-stage HCC have a median survival of approximately 16 months [32] [33]. TACE improves median survival to 19–20 months in RCTs [32] [33] and is considered the standard of care for these patients.

The selected nature of our LA population, with particular reference to BCLC B, makes any comparison with historical results after TACE impossible and in this view prospective randomized clinical trials are needed.

It is pretty clear, however, that survival figures of our BCLC A patients ineligible for resection or percutaneous ablation remain in the survival ranges described after potentially radical therapies in the international literature [32]. Similarly, the remarkable low prevalence of severe adverse events after LA even in BCLC B patients, associated to the relatively good long term survival suggests a genuine potential advantage of LA on TACE in homogeneous subset of patients.

As for resection and percutaneous ablation [1], also our series of HCC patients treated by LA median showed a consistent risk of tumour recurrence intrinsically related to these loco-regional procedures. In many enrolled patients, however, LA was only the first step of a multimodal sequence of therapies used to treat the tumour. This aspect and the high safety profile of LA probably explain the good survival profile of our patients despite the high incidence of local and distant recurrences. This discrepancy between outcome endpoints is frequent for HCC patients and this is why experts in this field suggest to use patient survival as primary endpoint [2] to evaluate treatment efficacy.

According to recent guidelines [1], many patients enrolled in our study would be candidates for first-line liver transplantation. With this in mind, one of the main findings of our study is that LA offers these patients the chance of a potentially curative alternative to transplantation, theoretically enabling organ saving. In this study, the 5-year survival rate for stage BCLC-A (and thus potentially transplantable) cases was about 40% (Figure 3): this seems to be much lower than the figure achievable with liver transplantation [34], but in actual fact, if we consider survival after the latter from the time of listing for LT, the 5-year intention-to-treat survival rate drops to 51% [35], making it comparable with the results we achieved with LA. In this perspective, allocation of BCLC A patients to LT or to other potentially radical therapies including LA, may be considered as a function of specific organ availability, waiting list time or age [36].

In our series, 33 patients ultimately underwent LT as second-line therapy. This policy is similar to the one adopted by Belghiti et al. [37] for patients undergoing first-line resection, reserving “salvage” LT for patients who have recurrent HCC or liver failure. The results of our study thus point to a sort of “extension” of the concept of salvage transplantation as a possible strategy not only after liver resection but also after other potentially radical therapies such as LA. A similar “extension” has been recently proposed for patients undergoing percutaneous thermal ablation [38].

BCLC early and intermediate stages includes a wide spectrum of potential liver diseases in terms of hepatic function (from Child A-5 to Child score B-9 patients). The results we obtained on uni- and multivariate analyses demonstrate that portal hypertension, Child-Pugh score and MELD score have little influence on survival of our patients undergoing LA. This finding is in contrast with other publications on percutaneous ablation or liver resection, in which these parameters had a clearly prognostic relevance to postoperative outcome [39] [40]. Moreover, liver decompensation signs such as ascites or hyperbilirubinemia have been recently suggested as contraindications to TACE [8].

The most interesting aspect of our study as regards BCLC stage, therefore, is that LA proved a safe and apparently effective therapy even for patients with a moderately impaired liver function who would be suboptimal candidates for resection, percutaneous ablation, and TACE [8]. Patient super-selection and the favourable pathophysiology of minimally invasive approaches may justify our findings, however, larger studies are needed to confirm such prognostic results in this therapeutic setting.

Judging from the results of our study, a serum albumin level <37 g/l and a serum α-fetoprotein level >400 g/l are parameters that can serve preoperatively as negative prognostic factors for the long-term outcome of LA, so they should be taken into account during the patient selection process in order to exploit LA to best effect in terms of patient survival. In conclusion, the results of our study suggest that LA is a safe and effective therapeutic option for HCC patients ineligible for liver resection and/or percutaneous ablation due to their tumour's characteristics (location, size and number of nodules) and/or liver mild to moderate impairment.

The present studies presents a main limitation as we did not address a specific ablation procedure, but a treatment strategy based on the laparoscopic approach as being able to overcome several of the intrinsic limits of resection and percutaneous ablation. Thus, any consideration about the superiority of RF over alcohol injection or microwave would not be consistent with the core message of our study. The main implication of our strategy proposal for HCC patients—unsuitable for resection or percutaneous ablation—is to avoid that these patients be considered immediately for liver transplantation.

The latter is a critical point since our proposal would spare a large number of organs now used to transplant patients with small HCCs and only moderately decompensated cirrhosis.

Although only randomized clinical trials can confirm which is the best first-line therapy for this particular category of HCC patients, the current drawbacks of TACE in patients with impaired liver function [8] and the shortage of organs for liver transplantation [1] [2] already support the use of LA as a potential effective therapeutic option for HCC in the current clinical practice.
